# Conserved generation of short products at piRNA loci

**DOI:** 10.1186/1471-2164-12-46

**Published:** 2011-01-19

**Authors:** Philipp Berninger, Lukasz Jaskiewicz, Mohsen Khorshid, Mihaela Zavolan

**Affiliations:** 1 Biozentrum, Universität Basel and Swiss Institute of Bioinformatics, Klingelbergstrasse 50-70, 4056 Basel, Switzerland; 2 EMBL Grenoble, 6 rue Jules Horowitz, 38042 Grenoble, France

## Abstract

**Background:**

The piRNA pathway operates in animal germ lines to ensure genome integrity through retrotransposon silencing. The Piwi protein-associated small RNAs (piRNAs) guide Piwi proteins to retrotransposon transcripts, which are degraded and thereby post-transcriptionally silenced through a ping-pong amplification process. Cleavage of the retrotransposon transcript defines at the same time the 5' end of a secondary piRNA that will in turn guide a Piwi protein to a primary piRNA precursor, thereby amplifying primary piRNAs. Although several studies provided evidence that this mechanism is conserved among metazoa, how the process is initiated and what enzymatic activities are responsible for generating the primary and secondary piRNAs are not entirely clear.

**Results:**

Here we analyzed small RNAs from three mammalian species, seeking to gain further insight into the mechanisms responsible for the piRNA amplification loop. We found that in all these species piRNA-directed targeting is accompanied by the generation of short sequences that have a very precisely defined length, 19 nucleotides, and a specific spatial relationship with the guide piRNAs.

**Conclusions:**

This suggests that the processing of the 5' product of piRNA-guided cleavage occurs while the piRNA target is engaged by the Piwi protein. Although they are not stabilized through methylation of their 3' ends, the 19-mers are abundant not only in testes lysates but also in immunoprecipitates of Miwi and Mili proteins. They will enable more accurate identification of piRNA loci in deep sequencing data sets.

## Background

Members of the Argonaute family of proteins have been identified as key players in small-RNA-guided silencing pathways. Of these, proteins of the Piwi clade are predominantly expressed in germ cells where they associate with a small RNA population distinct from that of miRNAs, as it has been shown initially for mammals and insects [1-4]. Some of the Piwi-associated small RNAs (piRNAs) are involved in regulating the expression of transposable elements, a function which is particularly important during genome activation in the germ line. Evidence for this specific role of piRNAs comes from the studies in y [5-7], zebrafish [8,9] and mouse [10-13]. Although Piwi-protein-associated small RNAs have also been discovered in worm (where they are called 21U RNAs) [14-17], their function in this species is less clear. Post-transcriptional silencing of retrotransposon transcripts is achieved by a piRNA amplification mechanism called ping-pong [6,18]. Briefly, piRNAs that are generated by a so far unidentified mechanism and are complementary to retrotransposon transcripts guide Piwi proteins to cleave these transcripts. The position of the Piwi-catalyzed cleavage is the bond between the nucleotides that have base-pairing interactions with nucleotides 10 and 11 of the primary piRNA. This cleavage defines the 5' end of a secondary piRNA that is generated from the transposon transcript. Because a very high proportion of piRNAs have a uridine (U) at the first position and because the complementarity between piRNAs and targets is expected to be nearly perfect, secondary piRNAs typically have adenosines at position 10, which base-pairs with the U at the first position of the piRNA [6,18]. After the 3' end of the secondary piRNA is generated through cleavage by a yet unknown nuclease and is subsequently 2'-*O*-methylated by the action of the Hen-1 methyltransferase [5,19,20], the secondary piRNA is loaded into a Piwi protein to guide the cleavage of a new primary piRNA precursor. This results in the production of piRNAs from the same loci from which the initial piRNAs were derived. Though the function of piRNAs has been characterized in detail in only a few species, evidence for Piwi protein-catalyzed cleavage and secondary piRNA production (a 10 nt overlap of the 5' end of piRNA sequences from opposite strands of the genome) has been provided for vertebrates [10], insects [6,18], flatworms [21], sea anemones and sponges [22], suggesting an early origin of the ping-pong mechanism.

In mouse, piRNAs are observed in the developing germ cells starting from the stage of G1-arrested gonocytes in the embryonic mouse up to the round spermatids in adult mouse [12]. Two classes of piRNAs, with somewhat different characteristic lengths have been described [12]: piRNAs which are ≈ 26 nucleotides (nt) in length are observed prior to meiosis, pre-natally and in the pre-pachytene stage, whereas a population of longer piRNAs appear to take over around the pachytene stage. Evidence for secondary piRNAs production has been found up to this point only in the prenatal and pre-pachytene piRNA populations, which associate with the Mili and Miwi2 proteins [10,12]. In contrast, pachytene piRNAs, which associate with both Mili and Miwi, are thought to consist solely of primary piRNAs [12,23]. After initial studies defined the characteristic length of piRNAs to be in the range of 23-32 nucleotides [3,4,10,12,13], larger than that of miRNAs, most studies of piRNAs extracted and analyzed sequence reads whose length was in this range. Longer potential intermediates and longer or shorter by-products of the amplification loop, that could arise during the processing steps that generate both 5' and 3' ends of piRNAs and could be in principle observed in the deep sequencing libraries have therefore not been captured in these studies. Because such products may shed light on the individual steps of piRNA production and piRNA-dependent targeting, we re-analyzed the few published libraries from mouse, rat and platypus that covered a broader range of small RNA sizes (15-36 nt). We further generated and analyzed an additional library of small RNAs from adult mouse testes.

## Results

### Signature of processing products at piRNA loci

**Figure 1:**
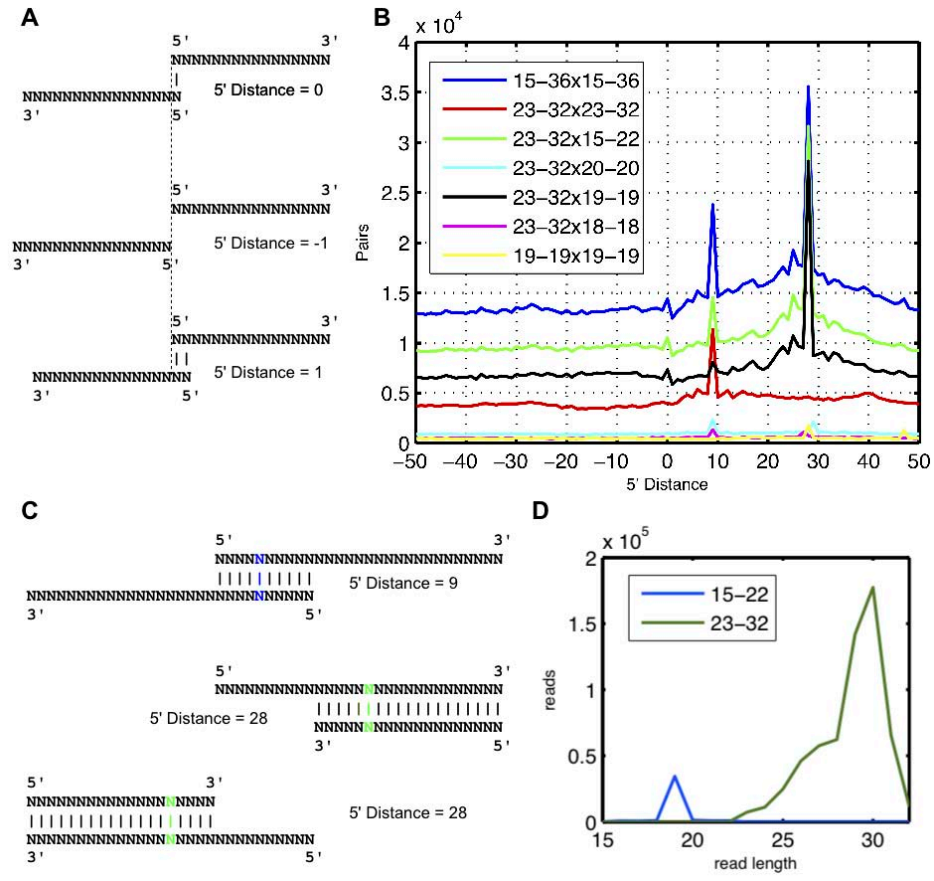
**Novel piRNA processing pattern involving short by-products of piRNA targeting**. (A) Sketch illustrating how the 5' distance of sequences from opposite strands was measured. (B) Frequencies with which various distances between the 5' ends of sequences derived from opposite chromosome strands occur in the mouse testes lysate data. On the x-axis, the 5' offset of sequences deriving from opposite strands is shown, and on the y-axis, the number of detected 'pairs'. The analysis was carried out for several subsets of sequences defined by the length of reads taken into account: blue line - all sequences of length 15-35 nt, red line - only sequences in the range of prototypical piRNAs (23-32 nt), green line - pairs were only counted if they involved on one strand a sequence in the range of piRNAs (23-32 nt) and on the opposite strand a sequence below that range (15-22 nt), black - as for green except that the short sequence had to be precisely 19 nt long. The peak at 9 (P9) is generated by sequences in the piRNA range, while the peak at 28 (P28) comes from pairs in which one sequence is long and the other is short, predominantly 19 nt. (C) Duplexes corresponding to the P9 (top) and P28 (bottom) patterns. The centers of the duplexes, which are used to analyze the co-occurrence of the two patterns are colored in blue and green respectively. (D) Histogram of the length distributions for the long (23-32 nt) and short (15-22 nt) sequences that contribute to the P28 pattern.

The library generated by Robine *et al. *[24] from adult mouse testes lysate, containing both pre-pachytene and pachytene piRNAs, is one of the few publicly available libraries of small RNAs that covers a relatively broad range of sequence lengths (15-36 nt). From the 9'936'151 sequences that mapped to the mouse genome, 6'144'650 sequences mapped uniquely and with no errors. We focused on these 6'144'650 sequences and determined genome-wide the relative distances between the 5' coordinates of small RNAs mapping to opposite strands of the chromosomes (see Methods). When two sequences from opposite strands have the same 5' coordinate, this distance is 0, whereas the primary and secondary piRNAs that are related through the ping-pong mechanism give a signature distance of 9 nucleotides (see Figure 1A). To avoid our results being dominated by a few very abundantly sequenced reads, we counted the pairs of sense-antisense sequences as follows. Given a sense read with copy number *c_s _*and an antisense read with copy number *c_a _*we count towards the distance *l_a _- l_s _*between the 5' end of the sense (*l_s_*) and antisense (*l_a_*) loci the smallest of the two copy numbers. For example, at a locus where we observed 3 sequences starting on the sense strand at position *l_s _*and 7 sequences on the antisense strand at position *l_a_*, we count 3 observed 'pairs' at distance *l_a _- l_s_*. An even more restrictive analysis, counting only the number of loci where a particular 5' distance occurred yielded the same results (see Additional file 1).

As expected, we detected a strong signal characteristic for piRNA-directed targeting (see Figure 1B), i.e. we found a relatively high frequency of sense-antisense pairs with a distance of 9 between the 5' ends of their loci. Surprisingly, we found that a 28 nt distance between 5' ends is also very common, though it has not been reported before. In total, about 1'010'500 of the uniquely, perfectly-mapping sequences in the library contributed to those two peaks (which is about 16% of all mapped sequences). Reasoning that previous studies that did not report the peak at 28 nt investigated relatively long sequences (longer than 22 nt), we split the sequence set based on the length of the small RNAs and repeated the analysis. Indeed, restricting the analysis to sequences longer than 22 nt (the piRNA range) revealed only the peak at 9 nt (P9). The peak at 28 (P28) only became apparent when we included sequences with a broader length distribution, because it corresponds to a pattern in which on one strand there is a long sequence, in the range of a prototypical piRNA, and on the opposite strand a shorter sequence (≤ 22 nt) (see Figure 1B). Furthermore, we found that the shorter sequence is almost always 19 nucleotides in length (see Figure 1B, C, D).

### P9 and P28 processing patterns co-localize

**Figure 2:**
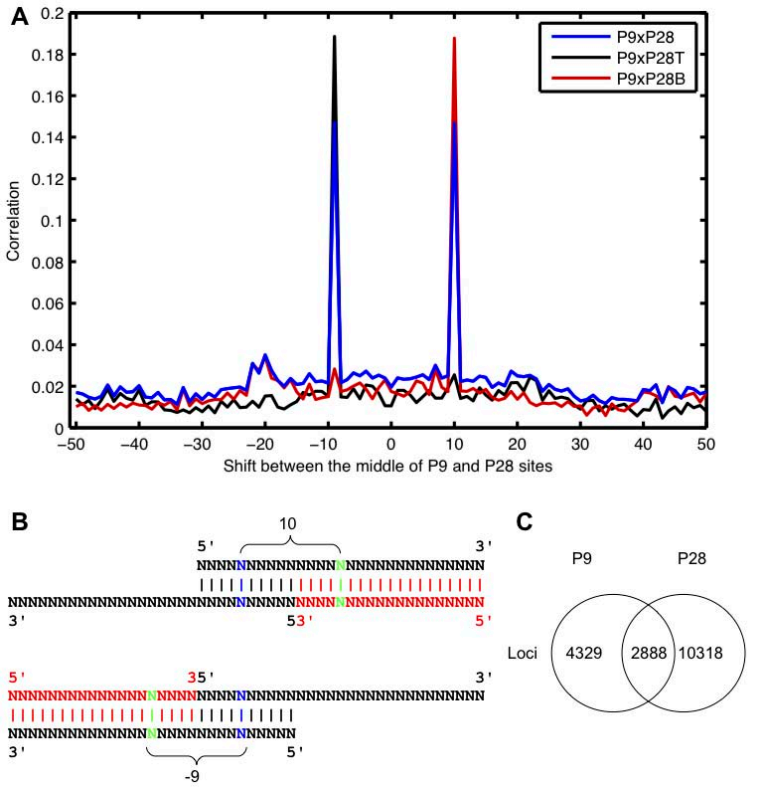
**P9 and P28 patterns co-localize**. (A) Cross-correlation analysis of the loci where P9 and P28 patterns occurred. Taking all sites together, two preferential locations of the centers are found (blue). By separating the locations of the peaks of 28 based on the strand on which the 19-mer contributing to the P28 pattern occurred, the two peaks were disentangled. For black we used P28 patterns in which the short sequence was found on the plus strand. For red we used P28 patterns in which the short sequence was found on the minus strand. (B) Triplex configurations that give rise to the co-occurring P9 and P28 peaks. (C) Venn diagram summarizing the co-occurrence of P9 and P28 processing patterns.

By inspecting some examples of pairs whose 5' ends were at a distance of 28 nt we noticed that in the duplex that would be formed by these sequences, the pairing between a 19 nt-long sequence and the long piRNA sequence would start at the 11*^th ^*nt from the 5' end of the long sequence, as if the 3' end of the 19 nt-long sequence were defined by the Piwi protein-dependent cleavage of the target RNA, coupled perhaps with the generation of the secondary piRNAs. Therefore, we decided to address systematically the question of whether the P28 and P9 processing patterns co-occurred. For this purpose we anchored the regions of overlap between sense and antisense pairs that gave rise to the P9 on one hand and the P28 peaks on the other hand at the middle of the interval defined by the 5' ends of the sense-antisense pairs (see Figure 1C). We then performed a genome-wide cross-correlation analysis of those positions (see Methods) and obtained two symmetrical peaks at -9 and 10, indicating that the middle of a P28 interval occurs preferentially either 9 nt downstream or 10 nt upstream of the middle of a P9 interval (see Figure 2A). By separately analyzing the cases in which the short sequence of a P28-defining pair occurred on the plus (P28T sites) and on the minus strands (P28B sites), we established that the peak at -9 corresponded to cases in which the short sequence was on the positive strand and the peak at 10 to cases in which the short sequence was on the negative strand, as expected from the sketch shown in Figure 2B. Thus, cross-correlation analysis reveals that sense-antisense pairs with a distance of 9 between 5' ends indeed co-occur with sense-antisense pairs with a distance of 28 between 5' ends. This configuration, shown in Figure 2B, is formed by two long piRNA sequences from opposite strands that overlap by 10 nucleotides, characteristic for the ping-pong mechanism, and a 19 nt-long sequence which is located upstream of one of the two piRNAs (see Figure 2B). From the 7'217 genomic loci, where the P9 pattern occurred and the 13'206 loci where the P28 pattern was detected, 2'888 loci had exactly this triplex (see Figure 2C).

### Short by-products are generated during piRNA targeting

To determine at what stage in piRNA biogenesis or targeting cascade the short products are generated, we retrieved the genomic sequence of all 2'888 loci from the mouse testes lysate data set where the two piRNAs together with the short by-product occurred. One of the main characteristics of primary piRNAs being their U bias [6,18] we determined the frequency of the U nucleotide at position 1 of the long piRNA sequences generated from these 2'888 loci. While the piRNAs that are located on the same strand as the 19-mers have a frequency of 44% U at position 1, the piRNAs on the opposite strand have a much higher frequency, namely 78%. This suggests that the 19-mer is a product that arises during piRNA-guided cleavage of target transcripts, whose 5' end is defined by a yet-unknown nuclease.

**Figure 3:**
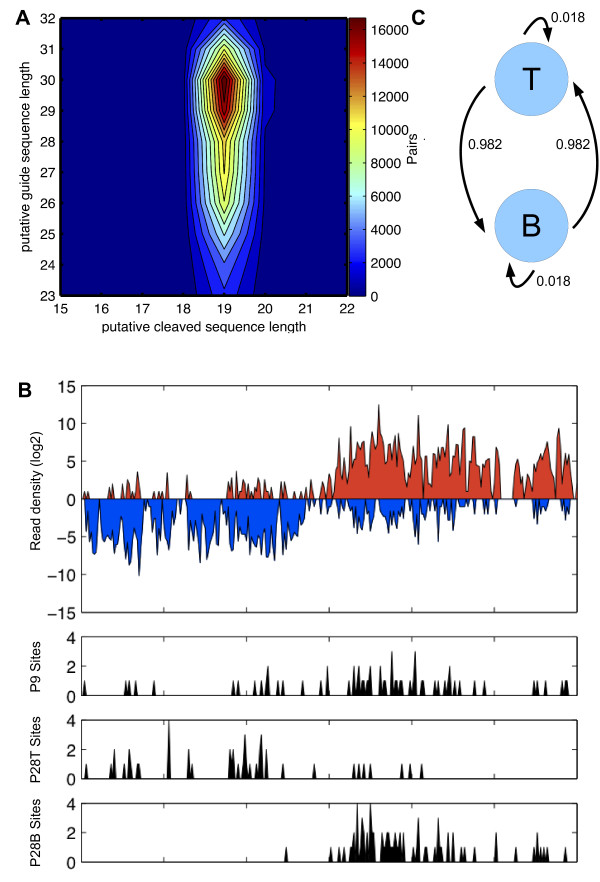
**Short by-products are generated during piRNA-directed targeting**. (A) Contour-plot of the frequency distribution of (piRNA length, by-product length) values. Cleavage products always have the 3' end located opposite position 11 of the guide piRNA. On the x-axis is the length of the cleavage product and on the y-axis the length of the guide sequence. (B) A 3 kb long region from chromosome 15 is shown (positions 59'105'450 to 59'108'450 on the mouse mm9 genome assembly). On the top, the log2 densities of unique mapping reads to the plus and minus strand within a 10 base window are shown in red and blue respectively. Below is the density of P9 patterns as well as P28 sites where the 19-mer is located at the plus and minus strand. (C) The Markov model with transition probabilities between P28T (T) and P28B (B) sites is shown.

To gain further insight into how the 5' end of the 19-mer processing product is generated, we examined the relationship between the size of short products whose 3' end appears to be defined by a piRNA-guided cleavage event and the size of the guiding piRNA. For this purpose we extracted all loci at which we observed a sense-antisense pair of sequences, with the 3' end of the shorter member of the pair being 10 nucleotides downstream of the 5' end of the longer sequence. We then recorded the lengths of the long and short member of the pair and generated a contour plot of the frequency of occurrence of different pairs of lengths. As shown in Figure 3A, while the sequence of the guide piRNA varies more widely in length, between 23 and 32 nt, the length of the short sequence is sharply peaked at 19 nt. Thus, it appears that the 5' end of the short processing product is defined with respect to the 5' end of the guide piRNA, rather than with respect to the length of the guide piRNA or of the duplex that this forms with its target. By inspecting regions where the P28 patterns occur, we noticed that within broader genomic regions, the 19-mers were preferentially located on one strand (see Figure 3B), contrary to what one would expect in a population of cells in which the ping-pong process is on-going. To systematically address the question of whether 19-mers occur interspersed on the two strands of a piRNA locus or show a strand bias, we built a simple Markov model with two states, T and B, corresponding to 19-mers on the plus strand (which we called P28T sites) and of those with the 19-mer on the minus strand (which we called P28B). We then estimated transition probabilities *p_T→T _*, *p_T→B_*, *p_B→T _*and *p_B→B _*from our data (see Methods). Here, the transition probability *p_T→B _*corresponds to the probability that a 19-mer on the bottom strand follows after a 19-mer on the top strand. If the P28 sites exhibit no strand bias, we expect similar transition probabilities *p_T→T _≈ p_T→B _≈ p_B→T _≈ p_B→B _≈ *0.5, whereas a strand-bias would result in a higher transition probabilities *p_T→T _*and *p_B→B_*, with *p_B→T _*and *p_T→B _*being smaller. From our data we estimated *p_T→T _*and *p_B→B _*to be 0.982, and *p_B→T _*and *p_T→B _*0.018. This suggests that in the sample that we analyzed it was typically one of the strands from a piRNA locus that generated the piRNA target and the 19-mers, not both as one may expect from the ping-pong model, even though secondary piRNAs were produced.

### Generation of short by-products is conserved across species

**Figure 4:**
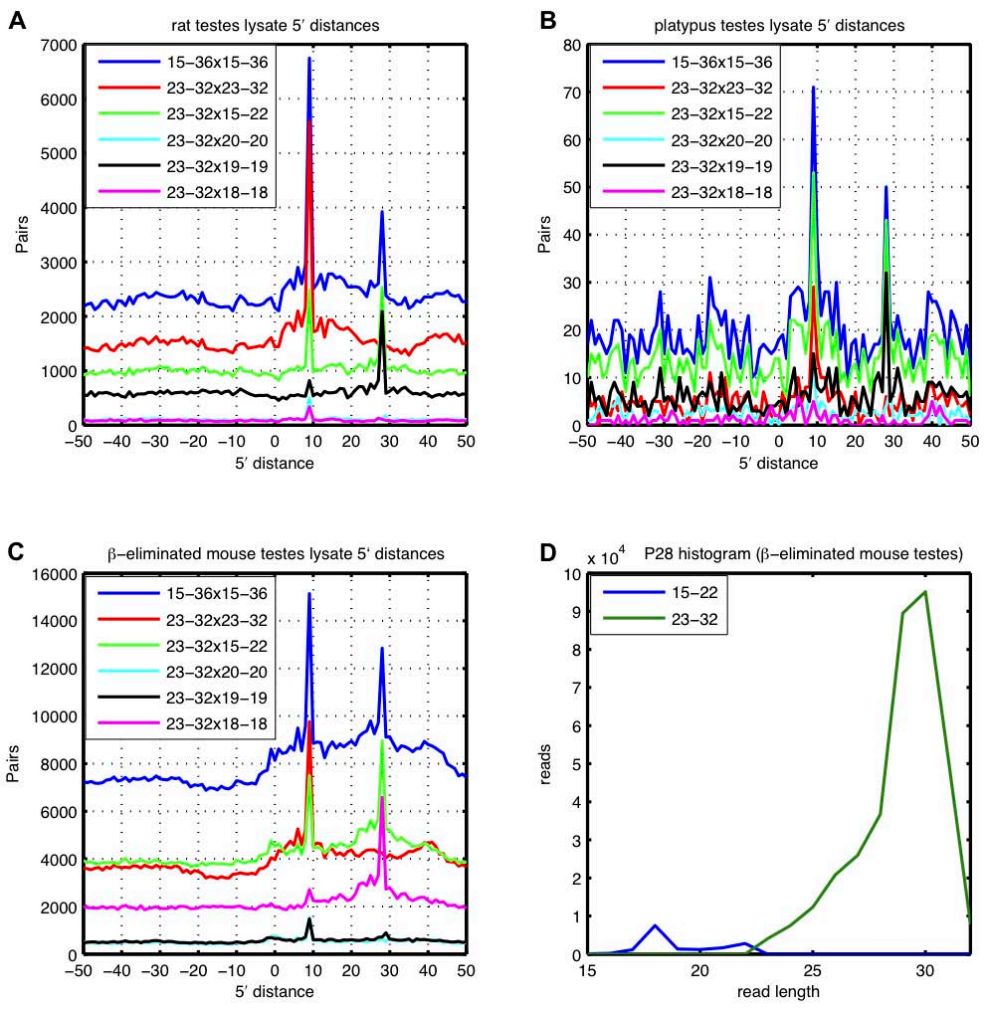
**The generation of short by-products is conserved across species**. Analysis of distances between 5' ends of overlapping sequences from opposite chromosome strands from rat (A) and platypus (B) testes lysates reveals the processing patterns that were already observed in mouse. (C) In mouse testes lysate treated with beta-elimination, 18-mer sequences contribute mostly to the P28 peak, suggesting that the by-products are not 3' methylated. (D) The histogram for the long (23-32 nt) and short (15-22 nt) sequences that contribute to the P28 pattern.

Reasoning that a meaningful processing pattern would be conserved across species, we analyzed publicly available testes lysate data sets from rat and platypus [25,26]. Indeed, we found evidence for the P28 processing pattern both in rat and platypus (see Figure 4A, B), suggesting that conserved enzymatic processes generate the short by-products in species ranging from platypus to mouse. On the contrary, we were not able to identify a P28 peak in the large data sets that were obtained from IPs of various proteins from the fly piRNA pathway [27] (Additional file 2), suggesting that the generation of 19-mers may be restricted to vertebrates. Furthermore, the rat data covered small RNAs not only from testes but also from brain, liver, kidney, spleen and heart, giving us the opportunity to determine whether the short processing products are testes-specific. Indeed, similarly to the P9 pattern, the P28 pattern was testes-specific (see Additional fie 3), as expected if these products were generated in the testes-specific piRNA pathway. Given that the 19-mers are sufficiently abundant for their capturing in sequencing samples and that the process that generates them is conserved across species, one may hypothesize that, similarly to piRNAs, they are stabilized and carry out an independent function. piRNAs are known to be stabilized through methylation of their 3' end [8], and we therefore wondered whether the 19-mers also carry this modification. To address this question, we incubated small RNAs from adult mouse testes with sodium periodate (NaIO_4_) and subjected them to *β*-elimination [1,28]. RNAs that contain both 2' and 3' hydroxyls react with NaIO_4 _and are shortened by one nucleotide during *β*-elimination. RNAs that have only one unmodified hydroxyl, like piRNAs that are 2'-*O*-methylated at their 3' end, do not react with NaIO_4_. Analysis of the *β*-eliminated data set revealed that the 19-mers appear to be shortened by the *β*-elimination process. Thus, the 19-mers are not stabilized through methylation of their 3' termini (see Figure 4C), and further studies will be required to establish whether or not they have a biological function.

## Discussion

It is unclear whether these 19-mers have a biological function or are simply by-products of piRNA biogenesis. If they were incorporated into Piwi proteins, they would allow the ping-pong mechanism to move on the transcript, instead of being limited to the location defined by the primary piRNA because the cleavage that would be guided by the 19-mer would occur at a different position in the transcript relative to the cleavage that is induced by the secondary piRNA located immediately downstream of the 19-mer. Interestingly, we did detect the P28 peak in a library constructed from Miwi protein immunoprecipitate [24], suggesting that 19-mers may associate with Miwi (Additional file 4A). However, it remains to be established whether they do so as guide RNAs or as by-products of piRNA targeting that are transiently associated with Piwi proteins. The P28 peak is not as clearly apparent in the library constructed from the immunoprecipitate of the Mili protein (Additional file 4B). We also find a much smaller number of loci with triplex configuration - primary piRNA, secondary piRNA and the 19-mer upstream of the secondary piRNA - in the Mili data (163) compared to the Miwi data (1'846), in spite of the fact that number of perfectly and uniquely mapping sequences that we analyzed was larger for Mili (7'976'050) compared to Miwi (6'517'925) (see also Table 1). This is unexpected, because previous studies implicated Mili rather than Miwi in secondary piRNA production in mouse [10,12]. Although 19-mers do appear to associate with Piwi proteins, the fact that they are not stabilized by 3' end methylation argues against their function as piRNAs. Their size and the apparent lack of the 3' end modification would allow them to load into Argonaute proteins and function in RNAi-like silencing as short interfering RNAs. Further analyses, including also immunoprecipitates of the Ago proteins from testis will be necessary for testing this hypothesis.

**Table 1:** Statistics about mapped sequences and processing sites

Library	total mappers	perfect unique mappers	P9 loci	P28 loci	Triplex loci
Lysate	9'936'151	6'144'650	7'217	13'206	2'888
Miwi-IP	9'989'850	6'517'925	8'187	6'518	1'846
Mili-IP	10'481'246	7'976'050	2'134	695	163
Lysate (*β*-eliminated)	25'464'790	11'799'063	6'511	4'263	1'064

This table summarizes the mapping statistics for the three libraries from Robine *et al. *[24] and the testes lysate library that we generated and then subjected to *β*-elimination.

## Conclusions

**Figure 5:**
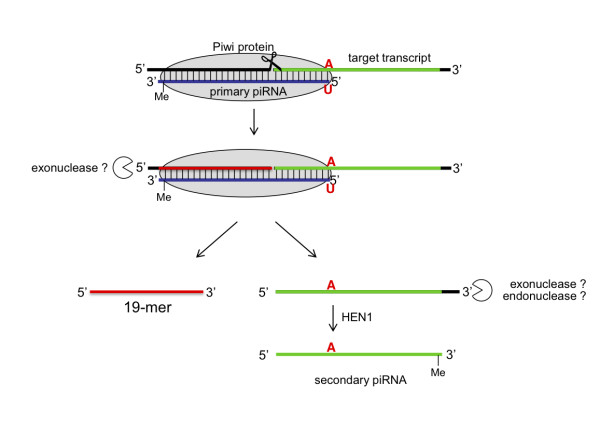
**Model of Piwi-protein-directed biogenesis of secondary piRNAs and 19 nt fragments**. Primary piRNA guides target cleavage and defines the 5' end of the secondary piRNA. 5' part of the target transcript that is not protected by the Piwi protein is trimmed by a 5'-3' exonuclease and the resulting 19-mer is formed.

A reanalysis of the piRNA sequence reads in three mammalian species surprisingly revealed that the signature of piRNA directed targeting and secondary piRNA generation is accompanied by 19 nt long sequences. Because these sequences occur at what appear to be Piwi-protein-mediated cleavage sites, we speculate that their 3' ends are generated during the same cleavage event that generates the 5' ends of the secondary piRNAs. Because the distance between the 5' end of the piRNA and the 5' end of the 19-mers is very precisely defined, our current hypothesis is that a 5'→3' exonuclease, which stops once it approaches the Piwi protein complex (that holds the piRNA-target duplex), is responsible for the formation of the 5' ends (see Figure 5).

Thus, the 19-mers that we identified as products of piRNA targeting yield new insights into this process. Furthermore, because we cannot currently define precisely what a piRNA is (other than using as criteria the length of the sequence and its association with the Piwi proteins), the frequently occurring P28 processing pattern can be used to identify loci where piRNA directed targeting takes place.

## Methods

### Sequence annotation

From the GEO database (http://www.ncbi.nlm.nih.gov/geo/) we obtained the following publicly available data sets that were generated in previous studies [24-27]: GSE10571 for platypus, GSE19054 for rat GSE19172 for mouse and GSE15186 for fly.

The data sets from rat were converted from Solid color space into sequence format with the script Solid2Solexa.pl (http://seqanswers.com/). After downloading the data sets, the adaptors were trimmed and all sequences of at least 15 nt in length were aligned against the corresponding genomes (excluding the mitochondrial chromosome) with oligomap [29]. For mouse, rat, platypus and fly, we obtained the genome assemblies mm9, rn4, ornAna1 and dm3 from the website of the University of California Santa Cruz (http://genome.cse.ucsc.edu).

After mapping the reads to the respective genomes, we only used those sequences that mapped uniquely (1 locus) and perfectly (without any error) for further processing analysis.

### Correlations of positions

We wanted to assess the correlation between the locations of the 5' ends of sequences deriving from opposite strands. We measured the distance Δ between the 5' ends for a given locus as follows:

(1) $$pairs{\left(\Delta \right)}^{+-}=\sum_{\forall i}\min(weight^{+}(i),weight^{-}(i+\Delta ))$$

where *weight*^+^(*i*) is the sum of the copy numbers of all of sequences that have their 5' end on the plus strand at a particular position *i *and *weight^- ^*(*i *+ Δ) is the sum of the copy numbers of all of sequences that have their 5' end on the minus strand at position *i *+ Δ. This measurement focuses only on the distance between the 5' ends and the length of the sequences is ignored. The 10 nucleotide overlap between the 5' ends of sequences from opposite strands that are generated by the ping-pong mechanism corresponds to Δ = 9. The results of this computation can be interpreted quite intuitively, it just indicates how often a particular distance has been observed in the entire set of reads. On the other hand, our measure is more conservative than that proposed by Olson *et al. *[30], who multiplied the counts of sequences overlapping from sense and antisense strands, thereby putting more weight on distances observed in genomic loci with a large number of reads.

For the systematic analysis of the relative position of the P9 and P28 peaks, we computed the cross-correlation between the genomic positions where the pairs of sequences contributing to these peaks occurred with the crosscorr function of Matlab. First, we identified the sequence reads that give rise to P28 and P9 patterns, respectively (see also Additional files 5 and 6). For each of these patterns, we generated independently a vector in which the index was the genomic location of the nucleotide that was located midway between the 5' end of the sequence on the plus strand and the 5' end of the sequence on the minus strand, and the entries were 1 if a pair was associated with a given genomic location, otherwise 0. We then applied cross-correlation analysis between the two vectors in a window of length 50. This resulted in two peaks, one at -9 and the other at 10. We then repeated this analysis using the same vector for P9, but two different vectors for P28. One of these vectors was constructed from pairs containing the 19-mer on the plus strand (P28T) and the other from pairs containing the 19-mer on the minus strand (P28B). With the first vector we obtained only the peak at -9 and with the second vector only the peak at 10.

### Strand-bias of processing sites

Assuming a Markov model with two states, T and B, and with transitions T→T, T→B, B→T, B→B, the most likely values of the transition probabilities can be estimated from the number of occurrences of T and B patterns at P28 patterns that occur consecutively along the chromosomes. For example, $p_{T\to T}=\frac{n_{T\to T}}{n_{T\to B}+n_{T\to T}}$.

### small RNA isolation, *β*-elimination and cloning

Total testis RNA from 6 months old C57/BL6 mice was isolated using TRIzol (Invitrogen) according to manufacturer's description. Sodium periodate treatment and *β*-elimination [28] were performed as described [1]. Briefly, 20 *μ*g of total RNA was incubated with freshly prepared NaIO_4 _(final concentration 25 mM) in borate buffer (30 mM borax, 30 mM boric acid, pH 8.6) for 10 min at room temp. Unreacted NaIO_4 _was quenched with glycerol and sample was dried under vacuum, resuspended in borax buffer (30 mM borax, 30 mM boric acid, 50 mM NaOH, pH 9.5) and incubated at 45°C for 45 min. RNA was precipitated with ethanol, 5' radiolabeled by T4 polynucleotide kinase (New England Biolabs) and resolved by 15% denaturing PAGE. Small RNA fractions sized between 15 and 40 nt were recovered from the gel, converted into a cDNA library as described [31] and Solexa sequenced. The deep sequencing data from this study have been deposited in the Gene Expression Omnibus (GEO) database, http://www.ncbi.nlm.nih.gov/geo (accession no. GSE26160).

## Authors' contributions

PB performed data analysis, LJ prepared the sequencing libraries and MK helped with the annotation of sequences obtained through deep sequencing. PB and MZ designed and coordinated the study. PB, LJ and MZ wrote the manuscript. All authors read and approved the final manuscript.

## Supplementary Material

Additional file 1 **Processing patterns occur at thousands of genomic loci**. On the x-axis, the 5' offset of sequences deriving from opposite strands is shown, and on the y-axis, the number of detected genomic loci. The analysis was carried out on the testis lysate data set from Robine *et al. *[24] for several subsets of sequences defined by the length of reads taken into account: blue line - all sequences of length 15-35 nt, red line - only sequences in the range of prototypical piRNAs (23-32 nt), green line - pairs were only counted if they involved on one strand a sequence in the range of piRNAs (23-32 nt) and on the opposite strand a sequence below that range (15-22 nt), black - as for green except that the short sequence had to be precisely 19 nt long. Click here for file

Additional file 2 **P28 processing patterns are not present in fly**. On the x-axis, the 5' offset of sequences deriving from opposite strands is shown, and on the y-axis, the number of detected genomic loci. The analysis was carried out on the piRNA libraries from Malone *et al. *[27] for several subsets of sequences defined by the length of reads taken into account. Although the P9 pattern was detectable in various libraries, no signal for the P28 pattern was detected. Click here for file

Additional file 3 **Processing is testes-specific**. Analysis of rat deep sequencing reads from various tissues [25] shows that the two processing patterns are testes specific. On the x-axis, the 5' offset of sequences deriving from opposite strands is shown, and on the y-axis, the number of detected pairs. The analysis was carried out for several subsets of sequences defined by the length of reads taken into account: blue line - all sequences of length 15-35 nt, red line - only sequences in the range of prototypical piRNAs (23-32 nt), green line - pairs were only counted if they involved on one strand a sequence in the range of piRNAs (23-32 nt) and on the opposite strand a sequence below that range (15-22 nt), black - as for green except that the short sequence had to be precisely 19 nt long. Click here for file

Additional file 4 **Miwi mostly contributes to the P28 processing pattern**. Re-Analysis of Miwi (A) and Mili (B) interacting small RNAs [24] suggests that Miwi mostly contributes to the P28 pattern. On the x-axis, the 5' offset of sequences deriving from opposite strands is shown, and on the y-axis, the number of detected pairs. The analysis was carried out for several subsets of sequences defined by the length of reads taken into account: blue line - all sequences of length 15-35 nt, red line -only sequences in the range of prototypical piRNAs (23-32 nt), green line - pairs were only counted if they involved on one strand a sequence in the range of piRNAs (23-32 nt) and on the opposite strand a sequence below that range (15-22 nt), black - as for green except that the short sequence had to be precisely 19 nt long. Click here for file

Additional file 5 **Sites_P28.txt**. This file contains the genomic coordinates and sequence information of the P28 sites formed by long sequences (23-32 nt) on one, and 19 nt sequences on the other strand. 1. Column: chromosome 2. Column: Strand of the 19-mer 3. Column: 5' Coordinate of the 19-mer 4. Column: 19-mer sequence with copynumber 5. Column: Strand of the long sequences 6. Column: 5' Coordinate of the long sequences 7. Column: Long sequences with copynumber. Click here for file

Additional file 6 **Sites_P9.txt**. This file contains the genomic coordinates and sequence information of the P9 sites formed by long sequences (23-32 nt). 1. Column: chromosome 2. Column: Strand of the top sequences 3. Column: 5' Coordinate of the top sequences 4. Column: Top sequences with copynumber 5. Column: Bottom strand 6. Column: 5' Coordinate of the bottom sequences 7. Column: Bottom sequences with copynumber. Click here for file
