# Supplementary figures and images for: Conserved generation of short products at piRNA loci
Source: BMC Genomics. 2011 Jan 19;12:46. doi: 10.1186/1471-2164-12-46 (PMC3037900; doi:10.1186/1471-2164-12-46)

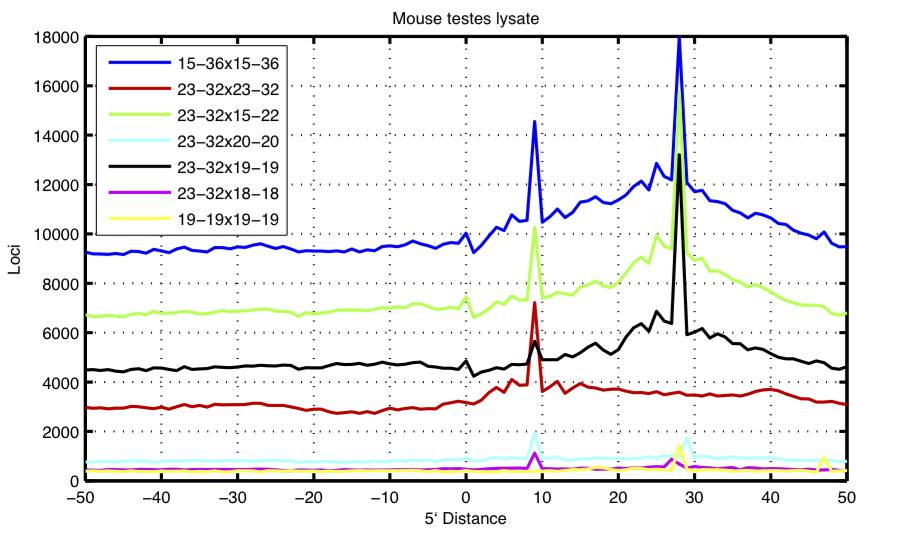

Supplement: Additional file 1 — Processing patterns occur at thousands of genomic loci. On the x-axis, the 5' offset of sequences deriving from opposite strands is shown, and on the y-axis, the number of detected genomic loci. The analysis was carried out on the testis lysate data set from Robine et al. [24] for several subsets of sequences defined by the length of reads taken into account: blue line - all sequences of length 15-35 nt, red line - only sequences in the range of prototypical piRNAs (23-32 nt), green line - pairs were only counted if they involved on one strand a sequence in the range of piRNAs (23-32 nt) and on the opposite strand a sequence below that range (15-22 nt), black - as for green except that the short sequence had to be precisely 19 nt long. [file 1471-2164-12-46-S1.PNG]

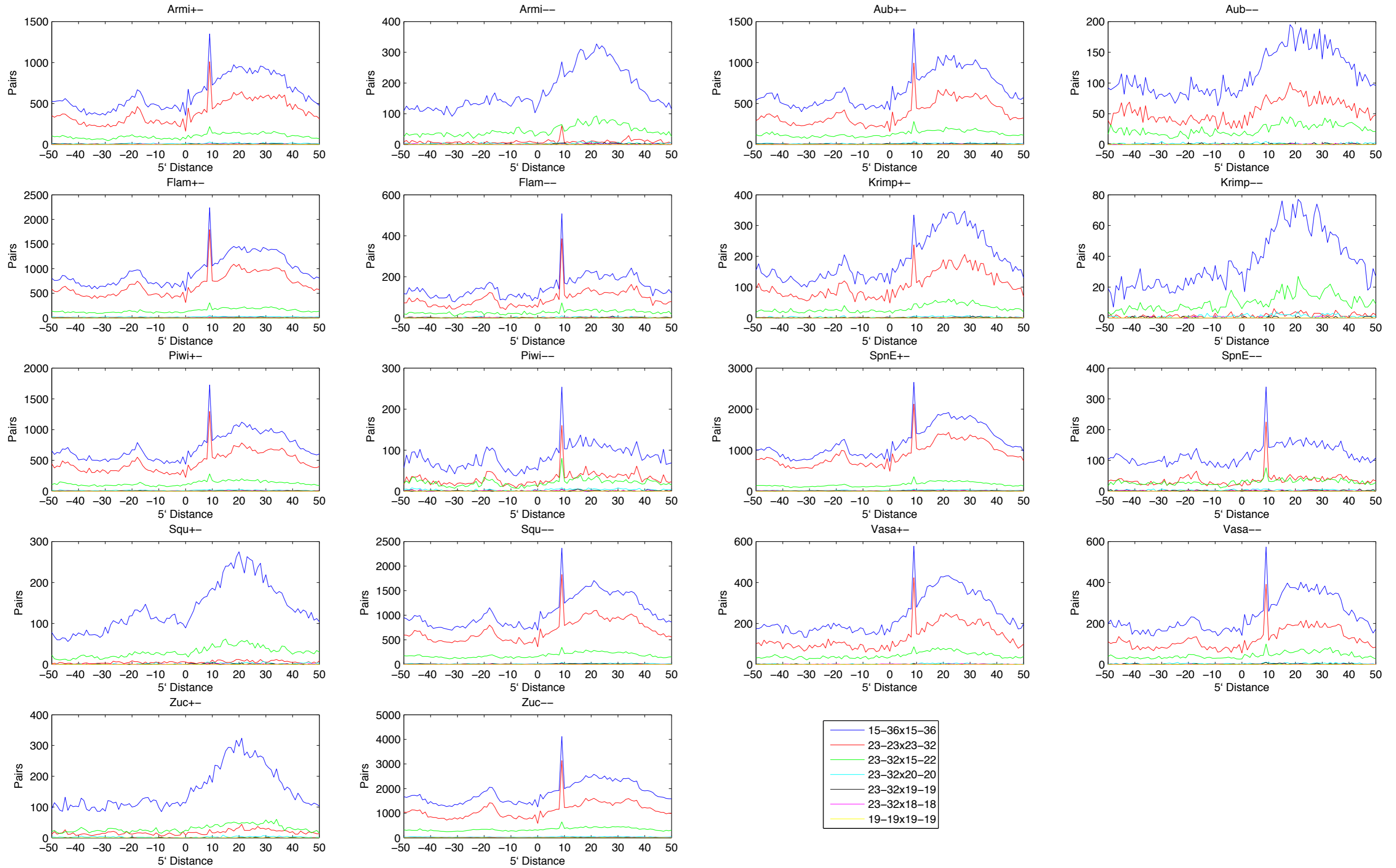

Supplement: Additional file 2 — P28 processing patterns are not present in fly. On the x-axis, the 5' offset of sequences deriving from opposite strands is shown, and on the y-axis, the number of detected genomic loci. The analysis was carried out on the piRNA libraries from Malone et al. [27] for several subsets of sequences defined by the length of reads taken into account. Although the P9 pattern was detectable in various libraries, no signal for the P28 pattern was detected. [file 1471-2164-12-46-S2.PDF]

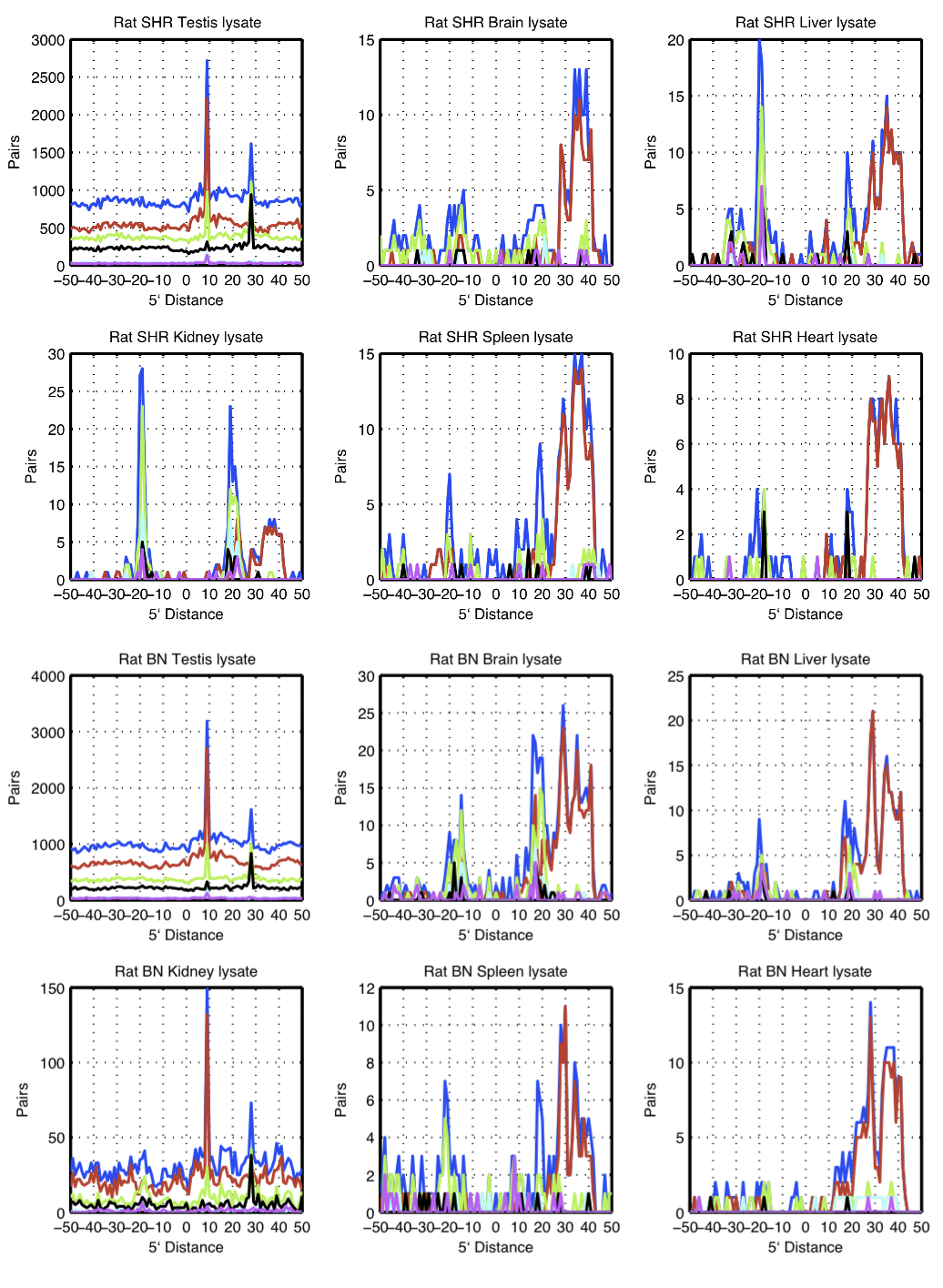

Supplement: Additional file 3 — Processing is testes-specific. Analysis of rat deep sequencing reads from various tissues [25] shows that the two processing patterns are testes specific. On the x-axis, the 5' offset of sequences deriving from opposite strands is shown, and on the y-axis, the number of detected pairs. The analysis was carried out for several subsets of sequences defined by the length of reads taken into account: blue line - all sequences of length 15-35 nt, red line - only sequences in the range of prototypical piRNAs (23-32 nt), green line - pairs were only counted if they involved on one strand a sequence in the range of piRNAs (23-32 nt) and on the opposite strand a sequence below that range (15-22 nt), black - as for green except that the short sequence had to be precisely 19 nt long. [file 1471-2164-12-46-S3.PNG]

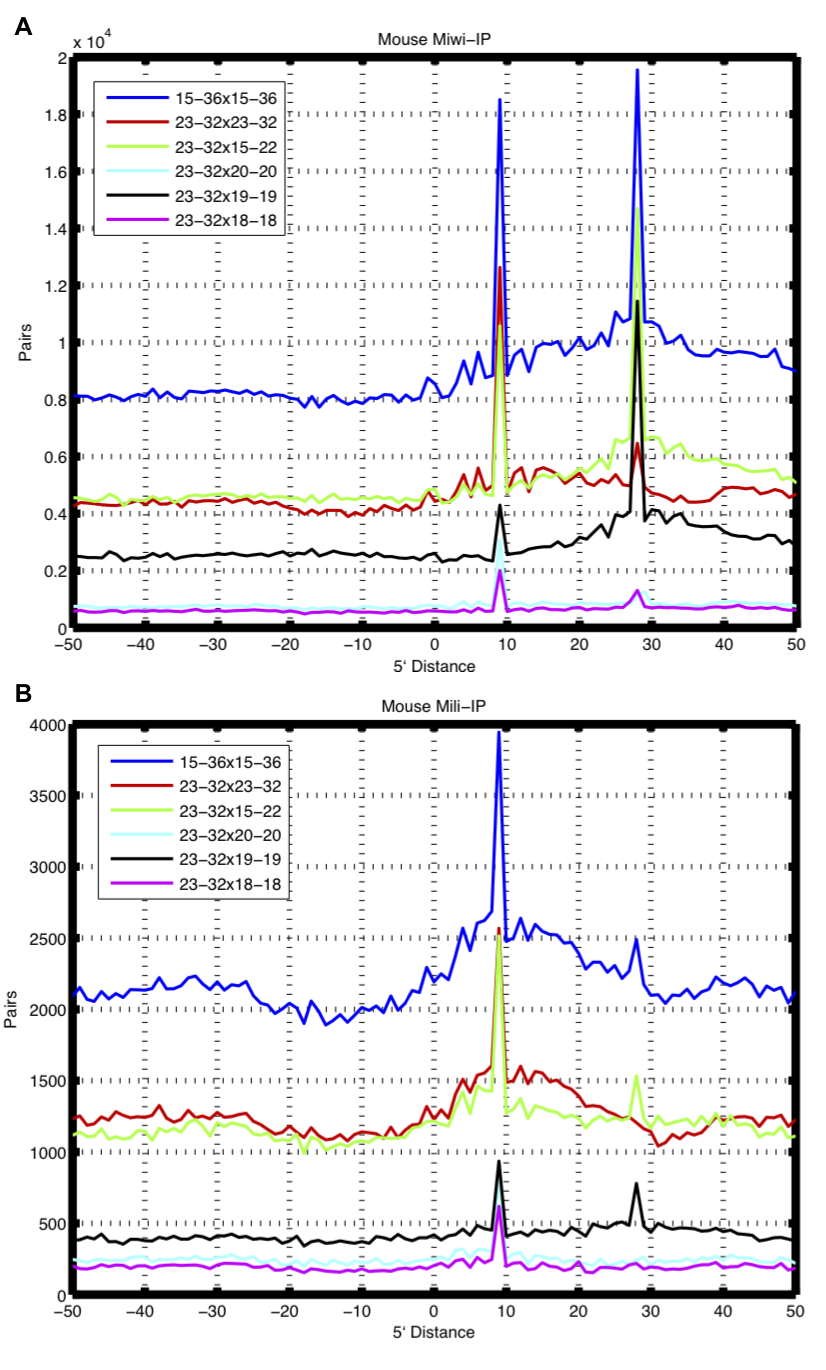

Supplement: Additional file 4 — Miwi mostly contributes to the P28 processing pattern. Re-Analysis of Miwi (A) and Mili (B) interacting small RNAs [24] suggests that Miwi mostly contributes to the P28 pattern. On the x-axis, the 5' offset of sequences deriving from opposite strands is shown, and on the y-axis, the number of detected pairs. The analysis was carried out for several subsets of sequences defined by the length of reads taken into account: blue line - all sequences of length 15-35 nt, red line -only sequences in the range of prototypical piRNAs (23-32 nt), green line - pairs were only counted if they involved on one strand a sequence in the range of piRNAs (23-32 nt) and on the opposite strand a sequence below that range (15-22 nt), black - as for green except that the short sequence had to be precisely 19 nt long. [file 1471-2164-12-46-S4.PNG]
